# [1-(Carboxy­meth­yl)cyclo­hexyl]­methan­aminium dihydrogen phosphate

**DOI:** 10.1107/S1600536810012973

**Published:** 2010-04-14

**Authors:** Syed Naeem Razzaq, Islam Ullah Khan, Onur Şahin, Orhan Büyükgüngör

**Affiliations:** a Materials Chemistry Laboratry, Department of Chemistry, GC University, Lahore 54000, Pakistan; b Department of Physics, Ondokuz Mayıs University, TR-55139 Samsun, Turkey

## Abstract

In the title salt, C_9_H_18_NO_2_^+^·H_2_PO_4_^−^, the cyclo­hexane ring is puckered, the total puckering amplitude *Q*_T_ being 0.555 (4) Å, and an intra­molecular N—H⋯O hydrogen bond generates an *S*(7) ring. In the crystal structure, inter­molecular N—H⋯O and O—H⋯O hydrogen bonds lead to *R*_2_^2^(14), *R*_3_^3^(8) and *R*_4_^2^(8) rings, generating a two-dimensional layer.

## Related literature

For related structures and medicinal background, see: Reece & Levendis (2008 ▶); Ibers (2001 ▶). For the graph-set analysis of hydrogen-bond patterns, see: Bernstein *et al.* (1995 ▶). For details of ring-puckering analysis, see: Cremer & Pople (1975 ▶). For bond-valence analysis and the positioning of H atoms, see: Brese & O’Keeffe (1991 ▶).
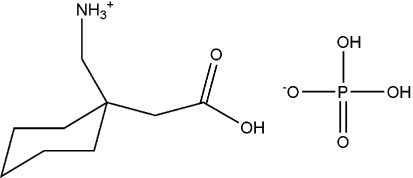


## Experimental

### 

#### Crystal data

C_9_H_18_NO_2_^+^·H_2_O_4_P^−^
*M* *_r_* = 269.23
Orthorhombic, 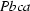

*a* = 10.473 (5) Å
*b* = 9.269 (3) Å
*c* = 26.468 (5) Å
*V* = 2569.4 (16) Å^3^
*Z* = 8
Mo *K*α radiation
μ = 0.23 mm^−1^
*T* = 296 K
0.31 × 0.25 × 0.22 mm

#### Data collection

Bruker Kappa APEXII CCD diffractometer
14659 measured reflections
3185 independent reflections
1853 reflections with *I* > 2σ(*I*)
*R*_int_ = 0.071

#### Refinement

*R*[*F* ^2^ > 2σ(*F* ^2^)] = 0.051
*wR*(*F* ^2^) = 0.155
*S* = 1.06
3185 reflections
178 parameters
6 restraints
H atoms treated by a mixture of independent and constrained refinement
Δρ_max_ = 0.34 e Å^−3^
Δρ_min_ = −0.45 e Å^−3^

### 

Data collection: *APEX2* (Bruker, 2009 ▶); cell refinement: *SAINT* (Bruker, 2009 ▶); data reduction: *SAINT*; program(s) used to solve structure: *SHELXS97* (Sheldrick, 2008 ▶); program(s) used to refine structure: *SHELXL97* (Sheldrick, 2008 ▶); molecular graphics: *ORTEP-3 for Windows* (Farrugia, 1997 ▶); software used to prepare material for publication: *WinGX* (Farrugia, 1999 ▶).

## Supplementary Material

Crystal structure: contains datablocks global, I. DOI: 10.1107/S1600536810012973/hb5398sup1.cif

Structure factors: contains datablocks I. DOI: 10.1107/S1600536810012973/hb5398Isup2.hkl

Additional supplementary materials:  crystallographic information; 3D view; checkCIF report

## Figures and Tables

**Table 1:** Hydrogen-bond geometry (Å, °)

*D*—H⋯*A*	*D*—H	H⋯*A*	*D*⋯*A*	*D*—H⋯*A*
O6—H6⋯O3^i^	0.83 (4)	1.77 (2)	2.602 (3)	173 (4)
O1—H1⋯O3^ii^	0.82 (2)	1.76 (2)	2.569 (3)	173 (5)
N1—H5⋯O1^iii^	0.88 (2)	2.26 (3)	2.929 (4)	133 (3)
N1—H5⋯O2^iv^	0.88 (2)	2.44 (3)	2.959 (3)	118 (3)
N1—H5⋯O5^iv^	0.88 (2)	2.47 (3)	3.065 (3)	125 (3)
N1—H4⋯O5	0.91 (2)	1.89 (2)	2.760 (4)	158 (3)
N1—H3⋯O4	0.90 (2)	1.86 (2)	2.752 (3)	174 (3)

Symmetry codes: (i) 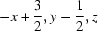; (ii) 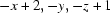; (iii) 
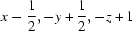
; (iv) 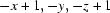.

**Fig. 1.**
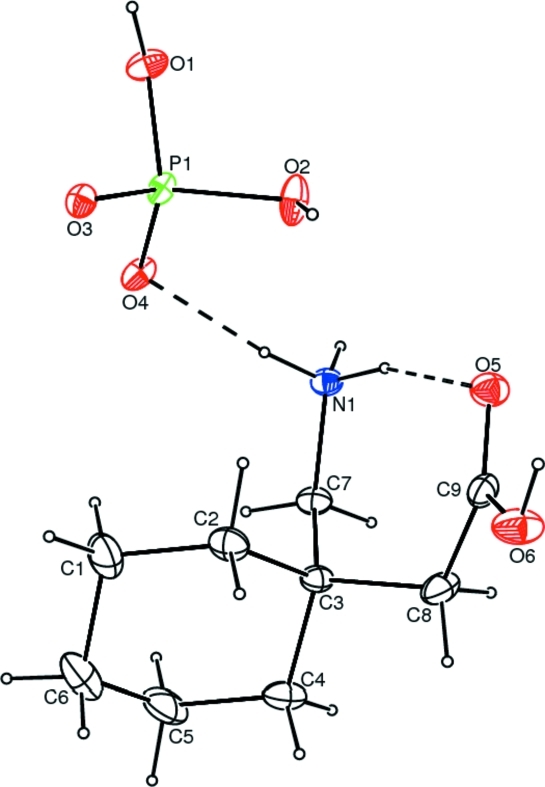
A view of one molecule of (I), showing displacement ellipsoids drawn at the 30% probability level. Hydrogen bonds are indicated by dashed lines.

**Fig. 2.**
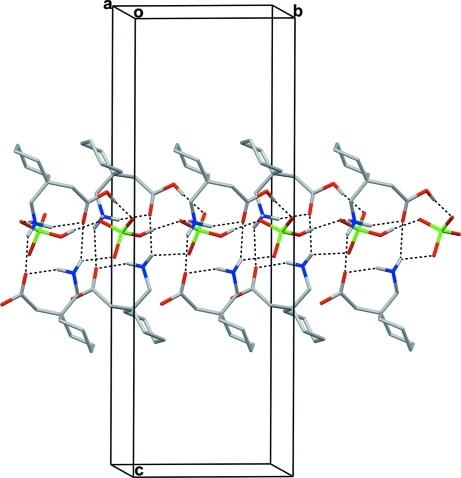
Part of the crystal structure of (I), showing the formation of a hydrogen-bonded sheet built from R33(8) and R42(8) rings. For the sake of clarity, H atoms not involved in the motif shown have been omitted.

## supplementary crystallographic information

### Comment

The title compound is a salt of gabapentin (Ibers, 2001; Reece & Levendis, 2008) an antiepileptic drug has potential application in treatment of neuropathic pain. Herein we report the synthesis and crystal structure of title compound (I).

The molecular structure and atom-labelling scheme are shown in Fig. 1. Selected bond distances and angles are given in Table 1. The C9—O6 bond length [1.310 (4) Å] indicate significant single-bond character, whereas the C9—O5 bond length [1.213 (3) Å] is indicative of significant double-bond character. The cyclohexane ring exhibits a puckered conformation, with puckering parameters (Cremer & Pople, 1975) q_2_ = 0.0246 (42) Å, q_3_ = 0.5544 (42) Å, Q_T_ = 0.5547 (42) Å, φ = 318 (10)° and θ = 1.81 (43)°. The O—P—O angles lie in the range 106.35 (14)–115.00 (12)°. Linkages P1—O1 and P1—O2 constitute POH groups, as confirmed both by the location of H atoms in the difference Fourier maps and by bond-valence calculations (Brese & O'Keeffe, 1991).

The atom N1 in the molecule at (x, y, z) acts as a hydrogen-bond donor (Table 2) to atom O5^iv^ so forming a centrosymmetric R_2_^2^(14) ring (Bernstein *et al.*, 1995) centred at (1/2, 0, 1/2). The combination of N—H···O and O—H···O hydrogen bonds generates R_3_^3^(8) and R_4_^2^(8) rings parallel to the [010] direction (Fig. 2).

### Experimental

To a 10 ml methanolic solution (0.002 M) of gabapentin was added 4 drops of phosphoric acid (85%). The mixture was heated and stirred for 30 min. Colourless prisms of (I) were obtained by slow evaporation from methanol.

### Refinement

All H atoms bound to C atoms were refined using a riding model, with C—H = 0.97 Å and U_iso_(H) = 1.2U_eq_(C) for methylene C atoms. Other H atoms bound to N and O atoms were located in difference maps and refined subject to a DFIX restraint of O—H = 0.82 (2) Å and N—H = 0.87 (2) Å.

### Crystal data

**Table:**

C_9_H_18_NO_2_^+^·H_2_O_4_P^−^	*F*(000) = 1152
*M**_r_* = 269.23	*D*_x_ = 1.392 Mg m^−^^3^
Orthorhombic, *P**b**c**a*	Mo *K*α radiation, λ = 0.71073 Å
Hall symbol: -P 2ac 2ab	Cell parameters from 1923 reflections
*a* = 10.473 (5) Å	θ = 3.0–21.6°
*b* = 9.269 (3) Å	µ = 0.23 mm^−^^1^
*c* = 26.468 (5) Å	*T* = 296 K
*V* = 2569.4 (16) Å^3^	Prism, colourless
*Z* = 8	0.31 × 0.25 × 0.22 mm

### Data collection

**Table:**

Bruker Kappa APEXII CCD diffractometer	1853 reflections with *I* > 2σ(*I*)
Radiation source: fine-focus sealed tube	*R*_int_ = 0.071
graphite	θ_max_ = 28.3°, θ_min_ = 1.5°
φ and ω scans	*h* = −13→7
14659 measured reflections	*k* = −11→12
3185 independent reflections	*l* = −35→35

### Refinement

**Table:**

Refinement on *F*^2^	Primary atom site location: structure-invariant direct methods
Least-squares matrix: full	Secondary atom site location: difference Fourier map
*R*[*F*^2^ > 2σ(*F*^2^)] = 0.051	Hydrogen site location: inferred from neighbouring sites
*wR*(*F*^2^) = 0.155	H atoms treated by a mixture of independent and constrained refinement
*S* = 1.06	*w* = 1/[σ^2^(*F*_o_^2^) + (0.0712*P*)^2^] where *P* = (*F*_o_^2^ + 2*F*_c_^2^)/3
3185 reflections	(Δ/σ)_max_ < 0.001
178 parameters	Δρ_max_ = 0.34 e Å^−^^3^
6 restraints	Δρ_min_ = −0.45 e Å^−^^3^

### Special details

**Table:**

Geometry. All esds (except the esd in the dihedral angle between two l.s. planes) are estimated using the full covariance matrix. The cell esds are taken into account individually in the estimation of esds in distances, angles and torsion angles; correlations between esds in cell parameters are only used when they are defined by crystal symmetry. An approximate (isotropic) treatment of cell esds is used for estimating esds involving l.s. planes.
Refinement. Refinement of *F*^2^ against ALL reflections. The weighted *R*-factor wR and goodness of fit *S* are based on *F*^2^, conventional *R*-factors *R* are based on *F*, with *F* set to zero for negative *F*^2^. The threshold expression of *F*^2^ > σ(*F*^2^) is used only for calculating *R*-factors(gt) etc. and is not relevant to the choice of reflections for refinement. *R*-factors based on *F*^2^ are statistically about twice as large as those based on *F*, and *R*- factors based on ALL data will be even larger.

### Fractional atomic coordinates and isotropic or equivalent isotropic displacement parameters (Å^2^)

**Table:**

	*x*	*y*	*z*	*U*_iso_*/*U*_eq_	
C1	0.6575 (4)	0.1582 (5)	0.67421 (15)	0.0667 (12)	
H1A	0.6569	0.2315	0.6481	0.080*	
H1B	0.7457	0.1334	0.6812	0.080*	
C2	0.5874 (3)	0.0253 (4)	0.65538 (12)	0.0413 (8)	
H2A	0.5967	−0.0514	0.6801	0.050*	
H2B	0.6266	−0.0070	0.6242	0.050*	
C3	0.4441 (3)	0.0522 (3)	0.64599 (10)	0.0301 (7)	
C4	0.3859 (4)	0.1206 (4)	0.69378 (12)	0.0477 (9)	
H4A	0.2983	0.1474	0.6866	0.057*	
H4B	0.3842	0.0485	0.7204	0.057*	
C5	0.4563 (4)	0.2527 (4)	0.71319 (15)	0.0667 (12)	
H5A	0.4489	0.3303	0.6888	0.080*	
H5B	0.4178	0.2847	0.7446	0.080*	
C6	0.5961 (5)	0.2189 (6)	0.72201 (16)	0.0874 (16)	
H6A	0.6039	0.1492	0.7492	0.105*	
H6B	0.6406	0.3061	0.7321	0.105*	
C7	0.4205 (3)	0.1584 (3)	0.60270 (11)	0.0329 (7)	
H7A	0.3293	0.1639	0.5966	0.040*	
H7B	0.4487	0.2533	0.6133	0.040*	
C8	0.3736 (3)	−0.0923 (3)	0.63691 (13)	0.0445 (9)	
H8A	0.3624	−0.1395	0.6693	0.053*	
H8B	0.2891	−0.0705	0.6239	0.053*	
C9	0.4354 (3)	−0.1977 (3)	0.60166 (12)	0.0343 (7)	
N1	0.4845 (2)	0.1225 (3)	0.55463 (9)	0.0296 (6)	
H3	0.5685 (19)	0.141 (4)	0.5561 (14)	0.067 (12)*	
H4	0.473 (4)	0.027 (2)	0.5469 (13)	0.069 (13)*	
H5	0.454 (3)	0.173 (4)	0.5291 (11)	0.066 (12)*	
O5	0.4571 (2)	−0.1736 (2)	0.55742 (8)	0.0417 (6)	
O6	0.4610 (3)	−0.3214 (3)	0.62334 (10)	0.0537 (7)	
H6	0.500 (4)	−0.373 (4)	0.6027 (13)	0.085 (15)*	
O1	0.8667 (2)	0.1053 (2)	0.47674 (8)	0.0366 (5)	
H1	0.929 (3)	0.066 (4)	0.4646 (16)	0.091 (16)*	
O2	0.7236 (2)	−0.0726 (2)	0.51893 (10)	0.0413 (6)	
H2	0.739 (5)	−0.149 (3)	0.5316 (16)	0.102 (18)*	
O3	0.92807 (17)	−0.00007 (19)	0.56151 (7)	0.0279 (5)	
O4	0.74292 (18)	0.17523 (19)	0.55177 (8)	0.0324 (5)	
P1	0.81774 (6)	0.05253 (7)	0.52950 (3)	0.0242 (2)	

### Atomic displacement parameters (Å^2^)

**Table:**

	*U*^11^	*U*^22^	*U*^33^	*U*^12^	*U*^13^	*U*^23^
C1	0.050 (2)	0.093 (3)	0.057 (2)	−0.009 (2)	−0.0153 (19)	−0.020 (2)
C2	0.0434 (18)	0.050 (2)	0.0308 (17)	0.0091 (16)	−0.0039 (14)	0.0055 (15)
C3	0.0353 (15)	0.0273 (16)	0.0277 (16)	0.0039 (13)	0.0068 (13)	−0.0017 (12)
C4	0.064 (2)	0.045 (2)	0.0339 (19)	0.0084 (18)	0.0157 (17)	−0.0015 (16)
C5	0.094 (3)	0.063 (3)	0.043 (2)	0.002 (2)	0.006 (2)	−0.0247 (19)
C6	0.101 (4)	0.106 (4)	0.055 (3)	−0.017 (3)	−0.016 (3)	−0.035 (3)
C7	0.0310 (15)	0.0361 (18)	0.0317 (16)	0.0113 (13)	0.0044 (13)	0.0009 (13)
C8	0.0446 (19)	0.0364 (19)	0.052 (2)	−0.0021 (15)	0.0225 (17)	−0.0022 (15)
C9	0.0320 (16)	0.0271 (17)	0.044 (2)	−0.0051 (13)	0.0077 (14)	−0.0016 (14)
N1	0.0291 (14)	0.0331 (16)	0.0265 (14)	0.0046 (12)	0.0017 (11)	0.0030 (12)
O5	0.0501 (14)	0.0350 (13)	0.0399 (14)	0.0027 (10)	0.0078 (11)	−0.0013 (10)
O6	0.0788 (18)	0.0353 (15)	0.0471 (15)	0.0119 (13)	0.0221 (14)	0.0026 (12)
O1	0.0325 (11)	0.0383 (12)	0.0391 (13)	0.0121 (10)	0.0096 (10)	0.0117 (10)
O2	0.0331 (11)	0.0184 (12)	0.0724 (17)	−0.0029 (9)	−0.0180 (11)	0.0081 (11)
O3	0.0233 (9)	0.0249 (10)	0.0356 (11)	0.0022 (8)	0.0001 (8)	0.0001 (9)
O4	0.0273 (10)	0.0182 (10)	0.0516 (13)	0.0048 (8)	0.0121 (9)	0.0033 (9)
P1	0.0193 (3)	0.0157 (4)	0.0377 (4)	0.0022 (3)	0.0025 (3)	0.0031 (3)

### Geometric parameters (Å, °)

**Table:**

C1—C2	1.519 (5)	C7—N1	1.476 (4)
C1—C6	1.526 (6)	C7—H7A	0.9700
C1—H1A	0.9700	C7—H7B	0.9700
C1—H1B	0.9700	C8—C9	1.498 (4)
C2—C3	1.541 (4)	C8—H8A	0.9700
C2—H2A	0.9700	C8—H8B	0.9700
C2—H2B	0.9700	C9—O5	1.213 (3)
C3—C7	1.531 (4)	C9—O6	1.310 (4)
C3—C4	1.541 (4)	N1—H3	0.897 (18)
C3—C8	1.549 (4)	N1—H4	0.912 (18)
C4—C5	1.519 (5)	N1—H5	0.881 (18)
C4—H4A	0.9700	O6—H6	0.83 (4)
C4—H4B	0.9700	O1—P1	1.566 (2)
C5—C6	1.515 (6)	O1—H1	0.817 (19)
C5—H5A	0.9700	O2—P1	1.548 (2)
C5—H5B	0.9700	O2—H2	0.799 (19)
C6—H6A	0.9700	O3—P1	1.513 (2)
C6—H6B	0.9700	O4—P1	1.502 (2)
			
C2—C1—C6	111.5 (3)	C5—C6—H6B	109.5
C2—C1—H1A	109.3	C1—C6—H6B	109.5
C6—C1—H1A	109.3	H6A—C6—H6B	108.1
C2—C1—H1B	109.3	N1—C7—C3	115.2 (2)
C6—C1—H1B	109.3	N1—C7—H7A	108.5
H1A—C1—H1B	108.0	C3—C7—H7A	108.5
C1—C2—C3	113.1 (3)	N1—C7—H7B	108.5
C1—C2—H2A	109.0	C3—C7—H7B	108.5
C3—C2—H2A	109.0	H7A—C7—H7B	107.5
C1—C2—H2B	109.0	C9—C8—C3	117.0 (2)
C3—C2—H2B	109.0	C9—C8—H8A	108.0
H2A—C2—H2B	107.8	C3—C8—H8A	108.0
C7—C3—C2	112.5 (2)	C9—C8—H8B	108.0
C7—C3—C4	106.6 (2)	C3—C8—H8B	108.0
C2—C3—C4	108.6 (3)	H8A—C8—H8B	107.3
C7—C3—C8	111.3 (3)	O5—C9—O6	123.0 (3)
C2—C3—C8	110.5 (3)	O5—C9—C8	124.2 (3)
C4—C3—C8	107.1 (2)	O6—C9—C8	112.7 (3)
C5—C4—C3	114.7 (3)	C7—N1—H3	111 (2)
C5—C4—H4A	108.6	C7—N1—H4	111 (2)
C3—C4—H4A	108.6	H3—N1—H4	109 (3)
C5—C4—H4B	108.6	C7—N1—H5	112 (2)
C3—C4—H4B	108.6	H3—N1—H5	107 (3)
H4A—C4—H4B	107.6	H4—N1—H5	107 (3)
C6—C5—C4	110.7 (3)	C9—O6—H6	109 (3)
C6—C5—H5A	109.5	P1—O1—H1	118 (3)
C4—C5—H5A	109.5	P1—O2—H2	118 (3)
C6—C5—H5B	109.5	O4—P1—O3	115.00 (12)
C4—C5—H5B	109.5	O4—P1—O2	107.84 (12)
H5A—C5—H5B	108.1	O3—P1—O2	110.23 (12)
C5—C6—C1	110.8 (3)	O4—P1—O1	106.50 (11)
C5—C6—H6A	109.5	O3—P1—O1	110.49 (12)
C1—C6—H6A	109.5	O2—P1—O1	106.35 (14)
			
C6—C1—C2—C3	55.9 (4)	C2—C1—C6—C5	−56.5 (5)
C1—C2—C3—C7	65.8 (4)	C2—C3—C7—N1	53.0 (4)
C1—C2—C3—C4	−51.9 (4)	C4—C3—C7—N1	172.0 (3)
C1—C2—C3—C8	−169.2 (3)	C8—C3—C7—N1	−71.5 (3)
C7—C3—C4—C5	−69.4 (4)	C7—C3—C8—C9	80.2 (3)
C2—C3—C4—C5	52.0 (4)	C2—C3—C8—C9	−45.5 (4)
C8—C3—C4—C5	171.4 (3)	C4—C3—C8—C9	−163.7 (3)
C3—C4—C5—C6	−54.9 (4)	C3—C8—C9—O5	−61.3 (4)
C4—C5—C6—C1	55.2 (5)	C3—C8—C9—O6	120.4 (3)

### Hydrogen-bond geometry (Å, °)

**Table:**

*D*—H···*A*	*D*—H	H···*A*	*D*···*A*	*D*—H···*A*
O6—H6···O3^i^	0.83 (4)	1.77 (2)	2.602 (3)	173 (4)
O1—H1···O3^ii^	0.82 (2)	1.76 (2)	2.569 (3)	173 (5)
N1—H5···O1^iii^	0.88 (2)	2.26 (3)	2.929 (4)	133 (3)
N1—H5···O2^iv^	0.88 (2)	2.44 (3)	2.959 (3)	118 (3)
N1—H5···O5^iv^	0.88 (2)	2.47 (3)	3.065 (3)	125 (3)
N1—H4···O5	0.91 (2)	1.89 (2)	2.760 (4)	158 (3)
N1—H3···O4	0.90 (2)	1.86 (2)	2.752 (3)	174 (3)

Symmetry codes: (i) −*x*+3/2, *y*−1/2, *z*; (ii) −*x*+2, −*y*, −*z*+1; (iii) *x*−1/2, −*y*+1/2, −*z*+1; (iv) −*x*+1, −*y*, −*z*+1.
